# Bryophyte cover and richness decline after 18 years of experimental warming in alpine Sweden

**DOI:** 10.1093/aobpla/plaa061

**Published:** 2020-11-24

**Authors:** Juha M Alatalo, Annika K Jägerbrand, Mohammad Bagher Erfanian, Shengbin Chen, Shou-Qin Sun, Ulf Molau

**Affiliations:** 1 Environmental Science Center, Qatar University, Doha, Qatar; 2 Department of Environmental and Biosciences, School of Business, Innovation and Sustainability, Halmstad University, Halmstad, Sweden; 3 Quantitative Plant Ecology and Biodiversity Research Lab., Department of Biology, Faculty of Science, Ferdowsi University of Mashhad, Mashhad, Iran; 4 College of Ecology and Environment, Chengdu University of Technology, Chengdu, China; 5 Institute of Mountain Hazards and Environment, Chinese Academy of Science, Chengdu, China; 6 Department of Biological and Environmental Sciences, University of Gothenburg, Gothenburg, Sweden

**Keywords:** Climate change, global warming, mosses, plant–climate interactions, plant litter, plant–plant interactions, species richness

## Abstract

Climate change is expected to affect alpine and Arctic tundra communities. Most previous long-term studies have focused on impacts on vascular plants, this study examined impacts of long-term warming on bryophyte communities. Experimental warming with open-top chambers (OTCs) was applied for 18 years to a mesic meadow and a dry heath alpine plant community. Species abundance was measured in 1995, 1999, 2001 and 2013. Species composition changed significantly from original communities in the heath, but remained similar in mesic meadow. Experimental warming increased beta diversity in the heath. Bryophyte cover and species richness both declined with long-term warming, while Simpson diversity showed no significant responses. Over the 18-year period, bryophyte cover in warmed plots decreased from 43 % to 11 % in heath and from 68 % to 35 % in meadow (75 % and 48 % decline, respectively, in original cover), while richness declined by 39 % and 26 %, respectively. Importantly, the decline in cover and richness first emerged after 7 years. Warming caused significant increase in litter in both plant communities. Deciduous shrub and litter cover had negative impact on bryophyte cover. We show that bryophyte species do not respond similarly to climate change. Total bryophyte cover declined in both heath and mesic meadow under experimental long-term warming (by 1.5–3 °C), driven by general declines in many species. Principal response curve, cover and richness results suggested that bryophytes in alpine heath are more susceptible to warming than in meadow, supporting the suggestion that bryophytes may be less resistant in drier environments than in wetter habitats. Species loss was slower than the decline in bryophyte abundance, and diversity remained similar in both communities. Increased deciduous shrub and litter cover led to decline in bryophyte cover. The non-linear response to warming over time underlines the importance of long-term experiments and monitoring.

## Teaser

This paper describes the effects of 18 years of experimental warming on bryophytes in a high alpine heath and a meadow community. Warming caused bryophyte cover to decrease by 75 % and 48 % in the heath and meadow, respectively, while richness declined by 39 % and 26 %. The decline in bryophyte cover was driven by a general decline in many species. Species loss was slower than the decline in bryophyte abundance. The decline in cover and richness first emerged after 7 years. Increased litter fall and increasing deciduous shrub cover led to a decline in bryophyte cover.

## Introduction

Arctic and alpine ecosystems are likely to experience a faster rate of warming than the global average (Chapin *et al.* 1995; Mack *et al.* 2004; IPCC 2013). Climate change is therefore likely to cause shifts in the range and relative abundance of Arctic/alpine organisms. Bryophytes in particular are predicted to be vulnerable to climate change, as many have low-temperature optima for photosynthesis and a narrow range of suitable temperatures for net photosynthetic gain (He *et al.* 2016). A long-term study (spanning the periods 1850–1939 and 1940–99) on the relative abundance of bryophytes based on biological collections across all major habitat types in Switzerland found that 16 species declined, four showed an increase and seven remained stable (Hofmann *et al.* 2007).

Bryophytes in Arctic and alpine regions are important in terms of biodiversity, typically exhibiting almost double the species richness of vascular plants in the Arctic (Matveyeva and Chernov 2000; Bahuguna *et al.* 2016; Mateo *et al.* 2016). Similarly, bryophytes are important contributors to cover and biomass (Longton 1984; Cornelissen *et al.* 2007). In term of biomass, bryophytes have been reported to contribute up to 91 % of above-ground biomass in a sedge-moss meadow in Western Taimyr, Siberia (Wielgolaski 1972), and an average of 38 % of above-ground biomass at a range of tundra sites (Wielgolaski 1972; Oechel and Sveinbjörnsson 1978). In a study in China, bryophyte cover was found to increase from 17.4 % to 95.6 % along an altitudinal transect from 2000 to 4200 m above sea level (a.s.l.) in the Gongga Mountains (Sun *et al.* 2013). In addition, bryophytes are important contributors to ecosystem services, while some host nitrogen-fixing bacteria and provide nitrogen inputs to ecosystems (During and Van Tooren 1990; Turetsky 2003). They also act as the major food source for some invertebrates (Collembola, cranefly, various species of Diptera) and vertebrates (Soay sheep, reindeer, barnacle geese) (Herbert and Prins 1982; Crafford and Chown 1991; Hodkinson *et al.* 1994; Smith *et al.* 2001; Glime 2006; Imada and Kato 2016). This is particularly common in cold environments (Herbert and Prins 1982). Despite this, vascular plants rather than bryophytes have been the focus of most climate change studies to date (Arft *et al.* 1999; Walker *et al.* 2006; Elmendorf *et al.* 2012a; 2012b; Alatalo *et al.* 2014b; Dumais *et al.* 2014; Wheeler *et al.* 2016; Zhang *et al.* 2016).

Most previous studies have shown that bryophyte biomass and/or cover is sensitive to long-term warming (8–20 years) at alpine and Arctic sites (Chapin *et al.* 1995; Wahren *et al.* 2005; Elmendorf *et al.* 2012a; Lang *et al.* 2012; Sistla *et al.* 2013). However, an increase in bryophyte cover has also been reported (Hudson and Henry 2010). Shorter-term studies (2–7 years) report contrasting results more frequently (Press *et al.* 1998; Jägerbrand *et al.* 2003; Bates *et al.* 2005; Klanderud 2008; Lang *et al.* 2009; Alatalo *et al.* 2014a; Koncz *et al.* 2018). The response of bryophytes to climate warming may also be context-specific, depending on potential competition from vascular plants (Molau and Alatalo 1998; Jägerbrand *et al.* 2012) and the origin of the sampled population, as shown in an *ex situ* experiment in Japan (Jägerbrand *et al.* 2014). Moreover, most studies provide summaries of cover/biomass of whole bryophyte communities (Sistla *et al.* 2013), while only a few have collected species-level data to study the impact on species or bryophyte diversity and richness (Molau and Alatalo 1998; Jägerbrand *et al.* 2003; Wahren *et al.* 2005; Klanderud 2008; Klanderud and Totland 2008; Lang *et al.* 2012; Alatalo *et al.* 2014a, 2015a; Sun *et al.* 2017). Climate change can also have indirect effects on bryophyte communities. Several studies have reported increasing shrubification of alpine and Arctic tundra ecosystems, a process that is predicted to increase in future due to climate change (Jägerbrand *et al.* 2009; Myers-Smith *et al.* 2011; Maliniemi *et al.* 2018; Myers-Smith and Hik 2018; Vowles and Björk 2019). Shrubification could potentially affect bryophyte communities, although previous studies have found inconsistent relationships between bryophytes and vascular plant abundance (Lang *et al.* 2012). Another question is whether species loss is delayed following a decline in abundance of bryophytes. It is not known whether species loss will occur rapidly, indicating that ‘rare’ species are lost initially, or at a longer interval after the decline in bryophyte abundance.

In the present study, bryophyte communities were examined following 18 years of experimental warming in two contrasting alpine sub-Arctic plant communities (mesic meadow and dry heath) in northern Sweden. The hypotheses tested were that: (i) bryophyte community composition is altered by long-term warming; (ii) bryophyte cover, richness and diversity are decreased by long-term warming; (iii) bryophyte cover, richness and diversity are negatively related to deciduous shrub cover and litter cover; and (iv) the negative impacts of warming are greater for mesic meadow, with its more developed vascular plant community, than for heath, with its sparser vascular plant community.

## Materials and Methods

### Study area

The study was conducted at Latnjajaure field station, which is located in the Latnjavagge valley (68°21′N, 18°29′E; 1000 m a.s.l.) in northern Sweden. The climate at the site is classified as sub-Arctic (Polunin 1951), with snow cover for 7–8 months of the year, cool summers and relatively mild, snow-rich winters. The growing season starts in late May and ends in early September (Molau *et al.* 2005). Mean annual air temperature in the study period (1993–2013) ranged from −0.76 to −2.92 °C (Alatalo *et al.* 2017a). Mean monthly temperature was highest in July, ranging from 5.9 °C in 1995 to 13.1 °C in 2013 (Alatalo *et al.* 2017a). Mean annual precipitation during the period was 846 mm, but in individual years it ranged from a low of 607 mm (1996) to a high of 1091 mm (2003) (Alatalo *et al.* 2017a). Climate data were collected throughout the year at the weather station at Latnjajaure field station, with hourly means, maxima and minima recorded (Molau and Alatalo 1998). Physical conditions in the valley soils vary from dry to wet, and from acidic to base-rich, with an associated variation in plant communities (Molau and Alatalo 1998; Lindblad *et al.* 2006; Björk *et al.* 2007; Alatalo *et al.* 2014b, 2017a). The mesic meadow community has a more well-developed vegetation cover (67 % canopy cover) (Alatalo *et al.* 2017), dominated by *Carex vaginata*, *C. bigelowii*, *Festuca ovina*, *Salix reticulata*, *S. polaris*, *Cassiope tetragona*, *Bistorta vivipara* and *Thalictrum alpinum* (Molau and Alatalo 1998; Alatalo *et al.* 2014b). The more sparsely vegetated heath community (54 % canopy cover) (Alatalo *et al.* 2017a) is dominated by *Betula nana*, *Salix herbacea* and *Calamagrostis lapponica* (Molau and Alatalo 1998; Alatalo *et al.* 2015b). Species richness and diversity in the heath and meadow experimental plots have been shown to be similar to those in the natural bryophyte communities in other vegetation types in the Latnjajaure area, such as dry heath, patterned heath, heath snowbed, mesic meadow, moist meadow and medium-rich fen (Jägerbrand *et al.* 2006).

### Experimental design and measurements

The 18-year study ranged from 1995 to 2013. Sampling in control and experimental (i.e. warming) plots was conducted in 1995, 1999, 2001 and 2013. At the start of the experiment, there were eight control plots and four plots with experimental warming in each plant community. However, not all initial control plots could be identified in 2013, so measurements were only made in four control and four experimental warming plots in each community in that year. Thus, for 2013, data from only four plots per treatment were used, while for 1995–2001, data from eight control plots were used. At the start of the experiment in July 1995, 12 plots (1 m × 1 m) with homogenous vegetation cover were marked out in both the alpine mesic meadow and the heath plant communities, and randomly assigned to treatments (control, experimental warming) in a pairwise design. Experimental warming was applied using hexagonal open-top chambers (OTCs), which were left in place on plots with warming treatment all year-around. In the initial years (1995–98), the temperature in the control and OTC plots was monitored for the entire year, in all 3 years, with Delta™ and Tinytag™ loggers. As found in other studies (Marion *et al.* 1997; Molau and Alatalo 1998; Hollister and Webber 2000), OTCs increased the air temperature by 1.5–3 °C compared with control plots with ambient temperature. It has also been shown that OTCs decrease canopy moisture (Hollister and Webber 2000), causing earlier snowmelt and prolonging the growing season (Molau and Alatalo 1998; Hollister and Webber 2000).

The species present in the plots **[see**  **Supporting Information—Table S1****]** were identified in the field or with the help of experienced bryophyte taxonomist Sven Franzén. Nomenclature for bryophyte species was retrieved from the literature (The Plant List 1.1 2013; USDA, NRCS 2020). Coverage of each bryophyte species was assessed using a 1 m × 1 m frame with 100 grid points (hereafter ‘hits’) (Walker 1996) in the middle of the growing season in 1995, 1999 (after 5 years), 2001 (after 7 years) and 2013 (after 18 years). Due to their hexagonal shape, the OTCs reduced the number of hits per plot to 77–87, and thus warmed plots had fewer hits than control plots. To enable comparison despite unequal sample size, the relative change over time in vegetation response for each treatment was calculated, as suggested by Kent (2011). The relative change was then used as the response variable in the statistical analyses (Kent 2011). To ensure accuracy and reproducibility, the same grid frame was used for each measurement, and fixed points at the corner of each plot allowed the frame to be replaced in the same positions within the plot on each measuring occasion. This method has been shown to be accurate in detecting changes in tundra vegetation (May and Hollister 2012).

### Statistical analyses

All statistical tests were conducted using R (R Core Team 2019).

### Species composition

#### Principal response curves.

 To test the hypothesis that bryophyte community composition is altered by long-term warming, we applied principal response curves (PRCs) (van den Brink *et al.* 2009) to the species composition (with bryophyte cover as an abundance measure) matrix data for the years 1995, 1999, 2001 and 2013. Because of unbalanced data for 2013, we opted not to conduct a formal permutation test. The *prc* function in the vegan package was used for this analysis (Oksanen *et al.* 2017; Oksanen 2018).

#### Analysis of similarity.

 To test whether the species composition in control and warming plots differed significantly in 1995 and 2013, we used analysis of similarity (ANOSIM) with 999 time permutations. We also tested the difference between 1995 and 2013 control and warming plots using ANOSIM. The species composition matrices used in ANOSIMs were the same as those of PRC. The differences between 1995 and 1999, and between 1995 and 2001, have been reported previously (reference anonymized). Separate ANOSIMs were performed for each type of vegetation. Therefore, eight ANOSIMs were performed. The *anosim* function in the vegan package was used for this analysis (Oksanen *et al.* 2017; Oksanen 2018).

### Species diversity and cover

To test the hypothesis that bryophyte cover, richness and diversity are decreased by long-term warming, we conducted the following three calculations and analyses.

### Bryophyte alpha diversity and cover

Bryophyte cover, species richness and diversity community parameters were calculated for comparison of warming and control plots in 1995–2013 for each vegetation type. From the point-frame data, the number of hits was summed up within each plot to produce plot-level abundance measures for each species. These values were used to calculate plotwise total bryophyte cover, species richness and Simpson’s diversity index *D* (Simpson 1949). Simpson’s diversity index was chosen since it is reliable even when the sample size is small (Mouillot and Lepretre 1999). In addition, it considers the diversity at the level of dominant species. The species richness metric considers the diversity of species without giving weight to the species (i.e. without considering their abundances), but Simpson’s diversity index (hereafter called ‘diversity’) gives higher weight to dominant species and rare species do not change its value. The calculated values were then transformed to relative change (ratio) for each individual plot for the whole period of the study (1995–2013), with 1995 data for each plot taken as the starting value. Relative change was used as the response variable because the number of hits per plot differed between treatments and because plots differed in their starting values of cover, richness and species composition. Data on bryophyte cover, species richness and diversity were checked for normality assumptions using Q–Q plots, and for homogeneity of variance using the Bartlett test. The Q–Q plots revealed that species richness, cover and diversity data were not normally distributed. Therefore, the Mann–Whitney *U*-test, a robust non-parametric test, was used to examine the effect of the experimental warming treatment on the relative change between all years in bryophyte cover, richness and diversity for the heath and meadow ecosystems. Friedman tests were used to compare these factors between years in each control and warming plot. Finally, boxplots showing changes in bryophyte cover, richness and diversity for the heath and meadow vegetation types were created using the ggplot2 package (Wickham 2009). These boxplots were calculated for both relative and absolute values, to better explain the variation in the data.

### Beta diversity

To test whether climate change affected beta diversity (i.e. variation in species composition in the plots) within treatments for the two community types studied, beta diversity was calculated for each year. To do so, a separate Hellinger distance matrix was created for control and warming plots in each year for each plant community, using the *vegdist* function in the vegan package (Oksanen *et al.* 2017; Oksanen 2018). The Hellinger distance approach was chosen because it is not affected by double zeros (Erfanian *et al.* 2019). The results were relativized with the same procedure as described above. The Mann–Whitney and Friedman tests were used for statistical comparisons. Boxplots showing variation in beta diversity for the heath and meadow vegetation types were created.

### Relationships between bryophyte richness and cover, deciduous shrub cover and litter cover

To assess changes in shrubification (here deciduous shrubs), which is hypothesized to have an impact on bryophyte communities, we used the same calculations and statistical tests as described above for bryophyte diversity and cover. To test the hypothesis that bryophyte cover and richness are negatively related to deciduous shrub and litter cover, we used multiple linear regression analysis. We regressed bryophyte species richness and cover against the cover of deciduous shrubs and litter and their interaction. We did not include site (i.e. meadow and heath), treatment (control and warming) and year as explanatory variables in the analysis, because they were confounder variables that affected both response variables (i.e. species richness and bryophytes cover) and explanatory variables (i.e. deciduous shrubs cover and litter cover) of this regression. 

## Results

### Species composition

#### Principal response curve.

**Figure 1.**
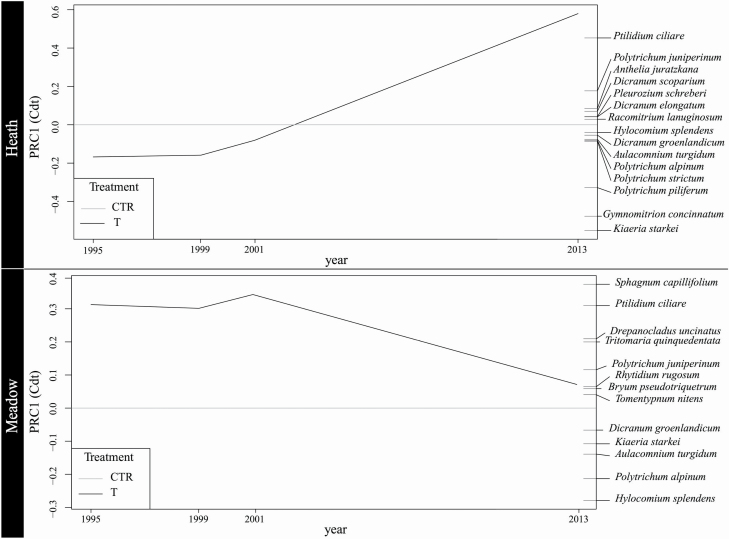
Principal response curve showing the effect of warming treatment over time on presence of bryophyte species in heath and meadow vegetation at Latnjajaure, northern Sweden. CTR = control plots; T = warmed plots (OTCs). The zero line indicates control plots.

The PRC for the heath and meadow vegetation types, with 1995 data (i.e. both control and warming plots) used as reference, showed the response over time of the communities to experimental warming. For the heath vegetation type, the species composition of control and warming plots was initially relatively similar and remained similar until 2001, after which differences emerged (Fig. 1). In PRC, species weights revealed the relative contribution of individual species to the community response. Species with positive weights increased more in the warming plots than in the control plots over time. In the heath vegetation type, *Ptilidium ciliare* showed an abundance increase in warming plots relative to control plots over time, whereas *Kiaeria starkei* and *Gymnomitrion concinnatum* abundance in warming plots was reduced compared with that in the control plots (Fig. 1). The PRC for the meadow, with 1995 used as reference, indicated that the species composition of the warming and control plots was relatively different at the start of the experiment, but between 2001 and 2013 it became more similar (Fig. 1). *Sphagnum capillifolium* and *P. ciliare* showed the greatest increase in abundance in warming plots compared with control plots, while *Hylocomium splendens* and *Polytrichum alpinum* showed the greatest decrease in abundance in warming plots compared with control plots (Fig. 1).

#### Analysis of similarity.

Analysis of similarities indicated that species composition had changed significantly in the heath (but not in the meadow) in both the control plots (*P* = 0.002) and warmed plots (*P* = 0.029) after 18 years, but there was no difference between control and warmed plots at the start or end of the study period in either heath or meadow (Table 1).

**Table 1.** Analysis of similarity results comparing differences in bryophyte species composition in control (CTR) and warming (Temp) plots in meadow and heath vegetation at Latnjajaure, northern Sweden, in 1995, 2013 and 1995 vs. 2013. Values in bold represent significance at 0.05 level.

Vegetation type	Year	Treatment	*P*-value	*R*
Meadow	1995	CTR vs. Temp	0.084	0.2353
Meadow	2013	CTR vs. Temp	0.386	0.02083
Meadow	1995 vs. 2013	CTR	0.521	−0.0294
Meadow	1995 vs. 2013	Temp	0.796	−0.156
Heath	1995	CTR vs. Temp	0.371	0.0275
Heath	2013	CTR vs. Temp	0.093	0.2812
Heath	1995 vs. 2013	CTR	**0.002**	0.6783
Heath	1995 vs. 2013	Temp	**0.029**	0.7344

### Bryophyte cover, richness and species diversity

**Figure 2.**
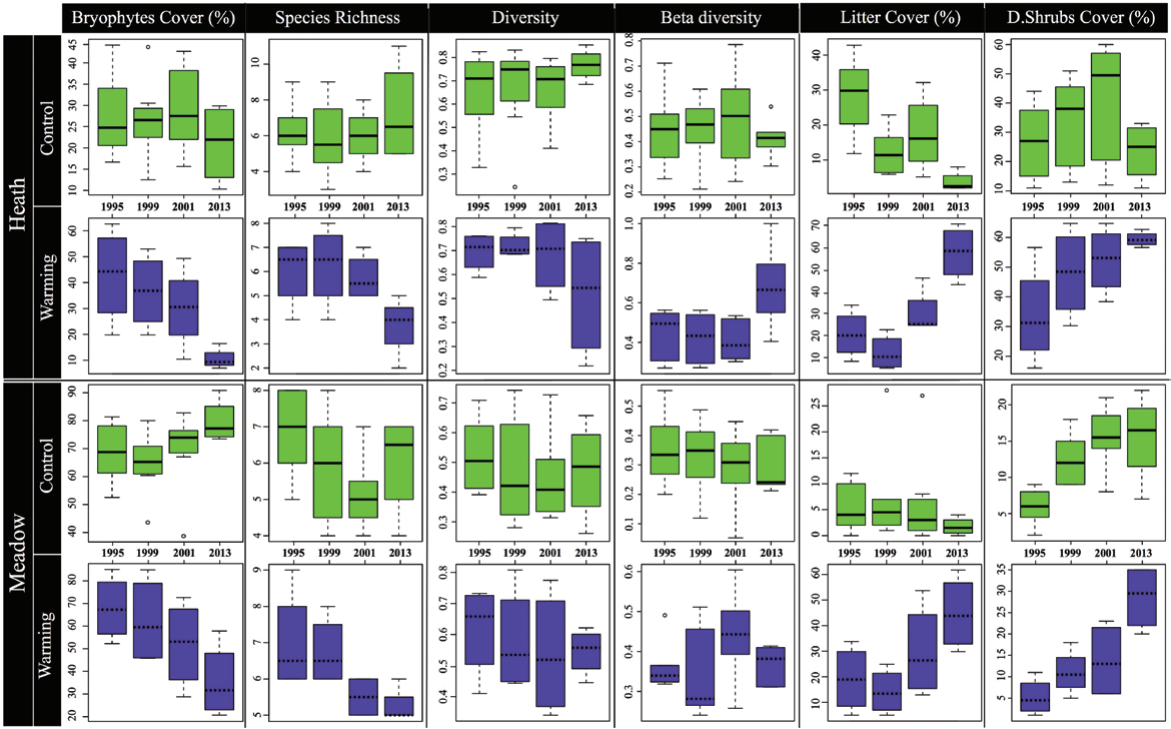
Boxplots showing bryophyte cover, bryophyte richness, bryophyte diversity, bryophyte beta diversity, litter cover and deciduous shrub cover over time in control (green, top row) and warmed (blue, bottom row) plots in heath (top panel) and meadow (bottom panel) vegetation at Latnjajaure, northern Sweden.

Bryophyte cover had declined by around 75 % in the heath and 50 % in the meadow after 18 years of experimental warming (Tables 2 and 3; Fig. 2: **see**  **Supporting Information—Fig. S1**). In contrast, these effects of warming were not significant after 7 years of treatments (Table 2). In the control treatments, bryophyte cover showed non-significant and inconsistent changes (Fig. 2).

**Table 2.** Probability (*P*) values in Mann–Whitney tests on the effects of warming on the measured variables. Values in bold numbers are statistically significant (α = 0.05). C = control; W = warming.

Vegetation type	Year	Bryophyte cover	Species richness	Diversity	Beta diversity	Litter cover	D. shrubs cover
Heath	1995	0.683	0.548	0.933	0.581	0.932	0.933
	1999	0.368	0.9	0.808	0.188	0.05	0.307
	2001	0.074	0.347	0.683	0.06	**0.008 (W)**	0.734
	2013	**0.029 (C)**	**0.028 (C)**	0.114	0.181	**0.029 (W)**	**0.029 (W)**
Meadow	1995	0.933	0.667	0.683	0.676	0.932	0.99
	1999	0.933	0.146	0.808	0.912	0.393	0.99
	2001	0.154	0.098	0.683	**0.029 (W)**	0.668	0.99
	2013	**0.029 (C)**	0.457	0.486	0.234	**0.029 (W)**	0.052

**Table 3.** Friedman test results on comparing response variables measured for control and warming plots between measurement years. Periods within brackets are significantly different from each other. Dec. = deciduous; ns = non-significant.

Vegetation type	Treatment	Measured variable	Friedman test + *post hoc* results
Heath	Control	Species richness	ns
Heath	Warming	Species richness	ns
Meadow	Control	Species richness	ns
Meadow	Warming	Species richness	ns
Heath	Control	Diversity	ns
Heath	Warming	Diversity	ns
Meadow	Control	Diversity	ns
Meadow	Warming	Diversity	ns
Heath	Control	Beta diversity	ns
Heath	Warming	Beta diversity	ns
Meadow	Control	Beta diversity	ns
Meadow	Warming	Beta diversity	(1995–2001)
Heath	Control	Bryophyte cover	ns
Heath	Warming	Bryophyte cover	(1995–2013)
Meadow	Control	Bryophyte cover	ns
Meadow	Warming	Bryophytes cover	(1995–2001)
Heath	Control	Dec. shrub cover	ns
Heath	Warming	Dec. shrub cover	(1995–99), (1995–2013)
Meadow	Control	Dec. shrub cover	(1995–99), (1995–2001), (1995–2013)
Meadow	Warming	Dec. shrub cover	(1995–2013)
Heath	Control	Litter cover	(1995–2013)
Heath	Warming	Litter cover	(1995–2013)
Meadow	control	Litter cover	ns
Meadow	warming	Litter cover	(1999–2001), (1999–2013)

Bryophyte richness declined significantly in response to experimental warming in the heath (*P* = 0.028), while it did not change significantly in the meadow (Table 2; Fig. 2: **see**  **Supporting Information—Fig. S1**). After 18 years of experimental warming, richness had declined by around 40 % in the heath and 25 % in the meadow (Fig. 2). Comparing the variation in relative values of species richness from 1995 to 2013 in both heath and meadow, warming plots showed a non-significant tendency for decreased species richness (Table 3; Fig. 2: **see**  **Supporting Information—Fig. S1**). The decline in bryophyte species richness accelerated after 2001 in both communities, but was not significant in the meadow (Fig. 2).

Although a decreasing trend was observed in warming plots (Fig. 2), bryophyte diversity did not show any significant response to experimental warming in either the heath or the meadow (Tables 1–3: **see**  **Supporting Information—Fig. S1**).

In the heath, there was a non-significant tendency for increased beta diversity in the warmed plots, and there were no significant differences between the years in either control or warming plots (Tables 2 and 3; Fig. 2: **see**  **Supporting Information—Fig. S1**). In the meadow, there was a significant difference in beta diversity between warmed and control plots in 2001 (Table 2), and warmed plots had significantly higher beta diversity 2001 than 1999 (Table 3; Fig. 2: **see**  **Supporting Information—Fig. S1**).

Litter cover in both heath and meadow plots showed contrasting responses in control and warming plots. Warmed plots in the heath had significantly higher litter cover in 2001 and 2013 (Table 2). In the heath, litter cover decreased significantly after 18 years in the control plots, while there was no change in the meadow (Table 3; Fig. 2: **see**  **Supporting Information—Fig. S1**). In warmed plots, litter cover significantly increased over time in both the heath (from around 22 to 58 % cover) and meadow (from around 19 to 45 %) (Tables 2 and 3; Fig. 2: **see**  **Supporting Information—Fig. S1**).

Deciduous shrub cover increased significantly in response to warming in the heath (Table 2; Fig. 2: **see**  **Supporting Information—Fig. S1**). No significant differences were observed between the years in control plots in the heath vegetation (Table 3). However, in warmed plots, deciduous shrubs increased over time (Table 3; Fig. 2). In the meadow, there was no significant difference between control and warmed plots (Table 2). However, in both control and warmed plots deciduous shrubs increased over time, with a ~300 % increase in shrub cover after 18 years in warmed plots (Table 3; Fig. 2: **see**  **Supporting Information—Fig. S1**).

### Relationships between bryophyte richness and cover, deciduous shrub cover and litter cover

**Figure 3.**
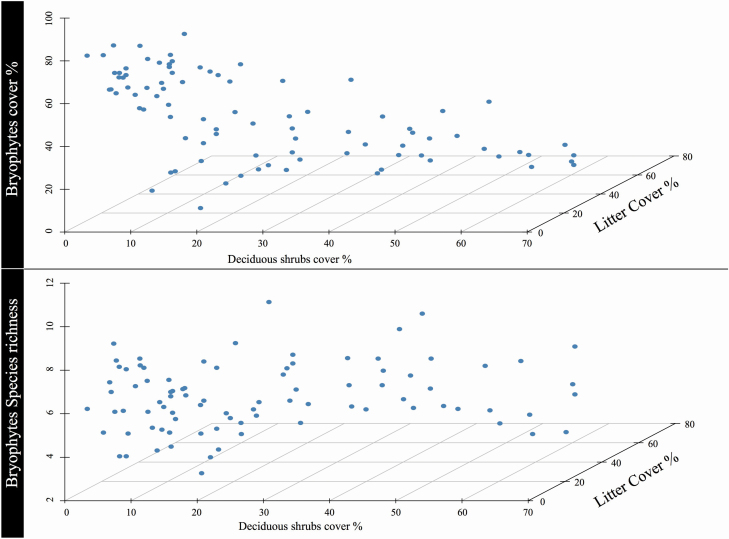
Three-dimensional scatter plots comparing the relationship between bryophyte cover and richness and deciduous shrubs and litter cover at Latnjajaure, northern Sweden.

In the multiple linear regression that was calculated to predict bryophytes cover based on the deciduous shrubs and litter cover, a significant regression equation was found (*F*(3, 84) = 36.69, *P*-value < 0.000), with an adjusted *R*^2^ of 0.552. The predicted bryophytes cover was equal to 77.883 − 0.774 (deciduous shrubs cover) − 1.112 (litter cover), where both deciduous shrubs and litter cover are measured in percent. Bryophytes cover decreased 0.774 % for each percent of deciduous shrubs cover and 1.112 % for each percent of litter cover. Deciduous shrubs cover increased the negative effects of litter cover with a coefficient of 0.013. Both explanatory variables and their interaction were significant, with higher values of bryophytes cover generally found in the lowest deciduous shrubs/litter cover values (Fig. 3).

The multiple linear regression for bryophyte species richness was significant. A significant regression equation was found (*F*(3, 84) = 3.279, *P* = 0.025), with an adjusted *R*^2^ value of 0.072. The predicted bryophyte species richness was equal to 5.717 + 0.028 (deciduous shrub cover) + 0.007 (litter cover), where both deciduous shrub and litter cover are measured in percent. Bryophytes species richness increased by 0.027 % for each 1 % increase in deciduous shrub cover and by 0.007 % for each 1 % increase in litter cover. Deciduous shrub cover decreased the positive effects of litter cover, with a coefficient of 0.001. Only the interaction was significant. However, although there was a significant regression, the *R*^2^ values showed that model performance was poor. The 3D graph revealed no observable pattern of species richness based on deciduous shrub and litter cover (Fig. 3).

## Discussion

This study examined the impact of 18 years of experimental warming on bryophyte communities in an alpine heath and an alpine meadow community in sub-Arctic northern Sweden. Most previous studies on the potential impact of climate change on bryophytes have focused on cover/biomass (Sistla *et al.* 2013), but this study we examined the potential impact of warming on bryophyte community composition. We determined changes in species composition, species richness and diversity, and assessed the potential impact of increased shrub encroachment and litter cover on bryophyte cover and richness. We found partial support for three of our four initial hypotheses. (i) As hypothesized, bryophyte community composition was altered by long-term warming; (ii) similarly to bryophyte cover, richness decreased under long-term warming, but the decline was smaller than the loss in cover and diversity was not affected; and (iii) deciduous shrub and litter cover had a negative effect on bryophyte cover, but not on bryophyte richness. However, in contrast to what was hypothesized, the negative impacts of warming were greater for the heath community compared to the meadow community.

### Changes in species composition in bryophyte communities under long-term warming

Principal response curve analysis showed that bryophyte species did not all respond to the experimental warming provided by OTCs in the same manner. This is in line with previous findings of both contractions and expansions in bryophyte species in Europe (Bergamini *et al.* 2009; Désamoré *et al.* 2012; Hodd *et al.* 2014). For example, a study on bryophytes using 10 521 specimens from biological collections in Switzerland found that 16 species had declined since 1850–1939, while four had increased (Hofmann *et al.* 2007). In the present study, we found that species composition of bryophytes in the meadow vegetation became more similar over time in the warming and control plots. In contrast, there were significant differences in species composition in the heath vegetation between control and warming plots. The hypothesis that bryophytes in mesic meadow are more vulnerable was not supported by the data. Instead, the results suggested that bryophytes in alpine heath vegetation are more susceptible to warming than those in meadow vegetation, supporting the suggestion that bryophyte communities may be less resistant in drier environments than in wetter habitats (Turetsky *et al.* 2012). It should be noted that PRC was initially developed for use in experiments such as ecotoxicology, where the control communities remain similar throughout the experiment, whereas the control plots in climate change experiments (i.e. this study) might experience large changes. Thus, while our PRC results show which species contributed to the difference (increase or decrease) between the warmed plots and control plots, it used the control plots as constants and placed them at a ‘zero’ line. Therefore, if the warming plots remain unchanged through the time and the control plots experienced compositional changes, the changes in the control plots (or plots experiencing ambient conditions) would appear as changes in the warmed plots. Consequently, it is not possible to know from the PRC whether the changes in species abundance displayed occurred due to changes in the warmed or the control plots. Experimental treatments are usually perceived as the cause of changes, but in natural systems ‘control’ (ambient) communities may change due to different factors, such as natural warming over the study period (Alatalo *et al.* 2017a), changes in precipitation between years (Fang *et al.* 2005), deposition of nutrients (Remke *et al.* 2009; Maskell *et al.* 2010) or changes in the communities due to natural succession/competition, etc. Here, we used ANOSIM and evaluated beta diversity changes to exclude the effects of this potential drawback on our interpretations. With this in mind, the PRCs are still useful in displaying differences between treatments over time and the species contributing to the changes.

The results showed that response patterns of beta diversity differed between sites, with the meadow community being more resistant to warming than the heath, and that plot-scale (i.e. small-scale) features are likely to play an important role in determining the resulting bryophyte communities. A study on alpine bryophyte communities in northern Italy showed that species track specific climate conditions along elevation gradients, leading to the prediction that climate change will increase species turnover of bryophyte communities, rather than leading to species loss (Nascimbene and Spitale 2017). In contrast, a study in the Canadian Rocky mountains found that bryophytes had a wide tolerance to temperature and elevation-related factors, thus having broader habitats and lower beta diversity along elevation gradients (Lee and La Roi 1979).

At our study site in northern Sweden, the shorter-term (1995–2001) (Alatalo *et al.* 2015a) and long-term (1995–2013) responses showed contrasting patterns. The latter underscores the importance of maintaining long-term monitoring and experimental studies to better understand community dynamics. Thus, the initial conclusions based on the fact that bryophytes did not show any significant changes failed to predict long-term responses correctly. In a previous study where we analysed bryophyte richness and diversity responses after 5 years of experimental warming and nutrient addition, we only found significant impacts for treatments with nutrient addition in the meadow, and no significant impact of the warming treatments (Jägerbrand *et al.* 2009). Consequently, the conclusions from our previous and present study clearly demonstrate that bryophyte communities show delayed responses, as shown for vascular plants. Many plant communities have been shown to be resistant to experimental perturbations during the first 10 years, but are increasingly affected thereafter (Komatsu *et al.* 2019).

### Species-specific responses

Bryophyte species can be expected to differ in their responses to warming, as they differ in their temperature optimum, desiccation tolerance and shading tolerance (Furness and Grime 1982; Glime 2006; Humbert *et al.* 2007; He *et al.* 2016). In order to understand why different species showed an increase (*P. ciliare* and *S. capillifolium*) or decrease (*K. starkei*, *G. concinnatum*, *H. splendens* and *P. alpinum*) in the PRCs, we consulted the BRYOATT list for Ellenberg values (light, moisture, nitrogen) (Hill *et al.* 2007) and broad temperature distribution (Hill and Preston 1998) for the species. This showed that the species with the largest decrease and largest increase in abundance did not differ markedly, with similar Ellenberg values for light (6 and 7), moisture (5, except *S. capillifolium* with 7) and nitrogen (1 and 2) found in the increasing and decreasing groups. Similarly, grouping within Arctic–alpine, sub-Arctic–sub-alpine and more boreal species (Hill and Preston 1998) did not explain the responses. This is line with the results obtained in the Canadian Rocky mountains, where bryophytes were shown to display a wide range of tolerance to elevation- and temperature-correlated factors (Lee and La Roi 1979). Therefore, it is likely that the species differ in relative competitive advantage in some other way, such as ability to cope with increasing litter deposition in warmed plots (Rincon 1988).

### Impacts of climate change on bryophyte cover, species richness and diversity

As hypothesized, bryophyte cover (in both communities) and richness (heath only) declined under long-term experimental warming, but the species loss was smaller than the decline in cover, indicating a delayed response in species loss. There was no significant effect on Simpson diversity. While the loss of species richness was already evident in the meadow in 2001, the decline in bryophyte cover and species richness increased after 2001 in both communities. In addition, although the decrease in bryophyte richness was larger in the heath, it was more delayed than the decline in the meadow community. This suggests that experimental warming may have caused rare species to become locally extinct earlier in the warmed plots of the meadow community compared with the warmed plots of the heath community. The hypothesis that bryophytes in mesic meadow are more vulnerable was not supported by the data. Instead, the PRC, cover and richness results suggested that bryophytes in alpine heath vegetation are more susceptible to warming than those in meadow vegetation, supporting the suggestion that bryophyte communities may be less resistant in drier environments than in wetter habitats (Turetsky *et al.* 2012). However, other studies have found polar and alpine bryophytes to be more negatively affected by experimental warming at wetter sites than at drier sites (Elmendorf *et al.* 2012a). This could be caused by increased drought stress at wetter sites as a result of experimental warming (Davey 1997; Turetsky 2003). For example, in the present experiment, long-warming in the meadow (but not in the heath) caused a decrease in soil moisture (Alatalo *et al.* 2017b).

Negative responses of bryophytes to experimental warming have also been reported in previous experimental studies (Elmendorf *et al.* 2012a; Lang *et al.* 2012; Sistla *et al.* 2013). Cover richness in the present study began to decline more markedly after 7 years of warming, as also found in other shorter-term studies in Sweden and Tibet (Alatalo *et al.* 2015a; Sun *et al.* 2017). However, bryophyte responses to long-term warming are not always negative (Van Wijk *et al.* 2003; Hudson and Henry 2010; Bokhorst *et al.* 2016). For example, 15 years of experimental warming resulted in an increase in bryophyte cover in High Arctic Canada (Hudson and Henry 2010). While bryophyte cover in our experimentally warmed plots declined in both the meadow and heath community, bryophyte cover tended to increase in the control plots in the meadow community, but not in the heath. This may be because Latnjajaure experienced natural warming of roughly 2 °C in the period 1993–2013, which may have caused a greater increase in vascular plant canopy in the heath community than in the meadow community (Alatalo *et al*. 2017a). Bryophytes are generally highly dependent on external water (He *et al.* 2016) and variations in annual rainfall can therefore potentially affect their photosynthesis and growth (Glime 2006; Jägerbrand *et al.* 2011). Annual precipitation varied substantially between years in the study period, but 2012 and 2013 did not have the highest or lowest annual precipitation (Alatalo *et al*. 2017a). Thus, it is unlikely that precipitation was the cause of changes in bryophyte cover in 2013. In addition, it is unlikely that the OTCs prevented colonization by bryophytes from outside the warmed plots, as a previous study in High Arctic Canada found that seed production in a wind-pollinated willow was not reduced by OTCs and that insect visitation was also unaffected (Robinson and Henry 2018). Thus, it is unlikely that the OTCs used in our study had a negative effect on wind-dispersed spores.

### Impact of shrubification and plant litter on bryophyte cover, species richness and diversity

Previous studies have reported an increase in deciduous shrub cover in alpine and Arctic tundra (Jägerbrand *et al.* 2009; Myers-Smith *et al.* 2011; Vowles *et al.* 2017; Maliniemi *et al.* 2018), Hence, we examined the correlation between this group of vascular plants and bryophytes. The increase in deciduous shrubs with warming is in line with previous predictions that increased temperature and nutrient mineralization will increase the productivity of vascular plants, which could have a negative effect on bryophytes (Molau and Alatalo 1998; Van der Wal *et al.* 2005). Our hypothesis of a negative correlation between deciduous shrub cover and bryophyte cover and richness was only partly supported by the data. While deciduous shrub cover had a negative impact on bryophyte cover, it had no impact on bryophyte richness.

We also examined the relationships between litter cover and bryophyte cover/richness. The results indicated a significant negative correlation between bryophyte cover (but not richness) and litter cover. This difference in the relationship between bryophyte cover and richness with deciduous shrub and litter may be because bryophyte species loss takes a longer time than bryophyte cover decrease. However, the differences in litter cover between control and warmed plots may also be an artefact caused by the constant presence of OTCs preventing litter from being blown away by the wind, and thus artificially increasing the litter cover. Previous studies have shown that bryophyte and lichen cover is negatively correlated with vascular plant canopy (Löbel *et al.* 2006; Pajunen *et al.* 2011; Jägerbrand *et al.* 2012; Alatalo *et al*. 2017a). Therefore, the widespread shrubification reported in alpine and Arctic tundra (Jägerbrand *et al.* 2009; Myers-Smith *et al.* 2011; Maliniemi *et al.* 2018; Myers-Smith and Hik 2018) could potentially have large impacts on cryptogam communities. However, a study using data from Latnjajaure, Sweden, and Toolik Lake, Alaska, found no negative relationship between bryophytes and abundance of vascular plants (Lang *et al.* 2012). As shrubification of alpine and Arctic tundra is expected to increase due to climate change, the effect of shrub encroachment on bryophytes needs to be monitored more closely in areas experiencing shrubification.

## Conclusions

Climate change is increasing at a more rapid rate than previously predicted, with widespread impacts on Arctic and alpine regions. This study showed that the important, but relatively understudied, Arctic and alpine bryophytes are likely to be adversely affected in the longer term. In this study, the responses to warming were non-linear over time, with negative effects accelerating after 7 years of experimental warming. Bryophyte cover declined more than richness, indicating a more delayed decline in species richness than in abundance. Bryophytes in the meadow community were expected to be more susceptible to warming, but PRC, cover and richness results indicated that the community in the drier heath habitat was more vulnerable. The decline in total bryophyte cover in both the heath and meadow communities was driven by a general decline in multiple species. Many of the most common species did not show any detectable changes, but the cumulative change was significant. Comparing the Ellenberg values for light, moisture and temperature optima of the bryophyte species experiencing the largest increase/decrease did not explain the different responses. Shrubification and indirect effects of litter (following shrubification) could be important mechanisms behind the decrease in bryophyte cover.

## Supporting Information

The following additional information is available in the online version of this article—

**Table S1.** Bryophyte species included in the community analysis (cover, richness and diversity).

**Figure S1.** Relative changes in richness, diversity, beta diversity, bryophyte cover, deciduous shrub cover and litter cover at start (1995), five (1999), seven (2001) and 18 years (2013) of warming treatment in heath and meadow vegetation at Latnjajaure, northern Sweden.

plaa061_suppl_Supplementary_Figure_S1

plaa061_suppl_Supplementary_Table_S1

plaa061_suppl_Supplementary_Data

## Data Availability

Data used for analyses are included in the electronic supplementary materials.

## Notes

**Populations & Communities. Chief Editor:** Jean Burns
