# Supplementary figures and images for: Bryophyte cover and richness decline after 18 years of experimental warming in alpine Sweden
Source: AoB Plants. 2020 Nov 24;12(6):plaa061. doi: 10.1093/aobpla/plaa061 (PMC7759949; doi:10.1093/aobpla/plaa061)

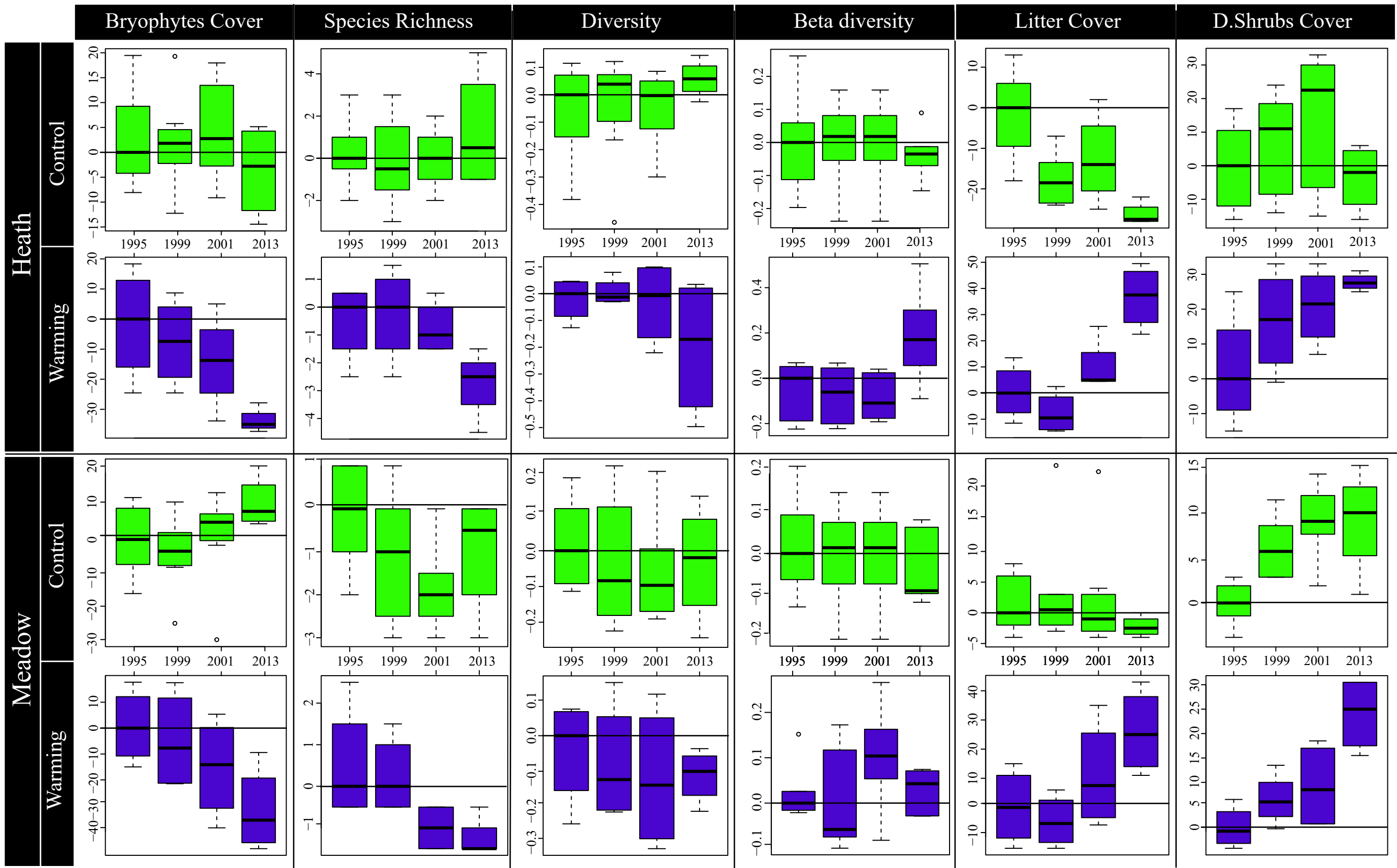

Supplement: plaa061_suppl_Supplementary_Figure_S1 [file plaa061_suppl_supplementary_figure_s1.pdf]
