# Supplementary material for: Bryophyte cover and richness decline after 18 years of experimental warming in alpine Sweden
Source: AoB Plants. 2020 Nov 24;12(6):plaa061. doi: 10.1093/aobpla/plaa061 (PMC7759949; doi:10.1093/aobpla/plaa061)
Supplement: plaa061_suppl_Supplementary_Table_S1 [file plaa061_suppl_supplementary_table_s1.pdf]

Supplementary table: Bryophyte cover and richness decline after 18 years of experimental warming in Alpine Sweden

*For correspondence. E-mail: alatalojm@gmail.com

Table S1. Bryophyte species included in the community analysis (cover, richness and diversity).

Species	
Anthelia juratzkana (Limpr.) Trevis.	*Lophozia grandiretis* (Lindb.) Schiffner
Barbilophozia kunzeana (Huebener) K. Müller	*Marsupella brevissima* (Dumort.) Grolle
Barbilophozia lycopodioides (Wallr.) Loeske	*Pleurocladula albescens* (Hook.) Grolle
Bryum pseudotriquetrum (Hedw.) P. Gaertn., B. Mey Scherb.	*Pohlia nutans* (Hedw.) Lindb.
Climacium dendroides (Hedw.) F. Weber & D. Mohr	*Pohlia nutans subsp. Schimperi* (Müll.Hal.)
Dicranum brevifolium (Lindb.) Lindb.	*Polytrichastrum alpinum* (Hedw.) G.L. Sm.
Dicranum elongatum Schleich. Ex Schwägr	*Polytrichum juniperinum* Hedw.
Dicranum fuscescens Turner	*Polytrichum piliferum* Hedw.
Dicranum groenlandicum Brid.	*Polytrichastrum sexangulare* (Flörke ex Brid.) G.L. Sm.
Dicranum scoparium Hedw.	*Ptilidium ciliare* (L.) Hampe
Gymnomitrion concinnatum (Lightf.) Corda	*Racomitrium lanuginosum* (Hedw.) Brid.
Gymnocolea inflata (Huds.) Dumort.	*Rhytidium rugosum* (Ehrh. ex Hedw.) Kindb.
Hylocomium splendens (Hedw.) Schimp.	*Sphagnum capillifolium* (Ehrh.) Hedw.
Kiaeria starkei (F. Weber & D. Mohr) I. Hagen	*Sphagnum warnstorfii* Russow
Leiocolea heterocolpos (Thed.) H. Buch	*Tritomaria quinquedentata* (Huds.) H. Buch
